# MicroRNA-409-3p Targeting at ATXN3 Reduces the Apoptosis of Dopamine Neurons Based on the Profile of miRNAs in the Cerebrospinal Fluid of Early Parkinson’s Disease

**DOI:** 10.3389/fcell.2021.755254

**Published:** 2022-01-10

**Authors:** Xuling Tan, Junjian Hu, Fengyu Ming, Lingling Lv, Weiqian Yan, Xinke Peng, Rongrong Bai, Qile Xiao, Hainan Zhang, Beisha Tang, Chunyu Wang, Jieqiong Tan

**Affiliations:** ^1^ Department of Neurology, The Second Xiangya Hospital, Central South University, Changsha, China; ^2^ Department of Medical Genetics, The Second Xiangya Hospital, Central South University, Changsha, China; ^3^ Center for Medical Genetics, School of Life Science, Central South University, Changsha, China; ^4^ Hunan Key Laboratory of Animal Models for Human Diseases, Central South University, Changsha, China; ^5^ Hunan Key Laboratory of Medical Genetics, Central South University, Changsha, China; ^6^ Department of Neurology, The First People’s Hospital of Huaihua City, HuaiHua, China; ^7^ Department of Neurology, Xiangya Hospital, Central South University, Changsha, China

**Keywords:** microRNAs, Parkinson's disease, cerebrospinal fluid—CSF, miR-409-3p, apoptosis

## Abstract

Precise recognition of early Parkinson’s disease (PD) has always been a challenging task requiring more feasible biomarkers to be integrated to improve diagnostic accuracy. MicroRNAs (miRNAs) of cerebrospinal fluid (CSF) are believed to be potential and promising candidate biomarkers for PD. However, the role of altered miRNAs of CSF play in PD is unclear. Here, we recruited patients with early stages of PD and controls to analyze the expression of miRNA in CSF by the Next Generation Sequencing (NGS). Furthermore, we tested the levels of these miRNA in SH-SY5Y cells treated with MPP^+^ using real-time quantitative PCR. We found 21 miRNAs were upregulated in CSF of early PD patients and miR-409-3p, one of the identified 21 miRNAs, was further confirmed in SH-SY5Y cells treated with MPP^+^. Also, more cells survived in the overexpression of the miR-409-3p group when SH-SY5Y cells and mice were treated with MPP^+^ and MPTP, respectively. Mechanistically, we demonstrated the binding of miR-409-3p and 3’UTR of ATXN3 through a dual luciferase reporter gene assay. Moreover, miR-409-3p mimic reduced the aggregation of polyglutamine-expanded mutant of ATXN3 and apoptosis. Our results provide experimental evidence for miR-409-3p in CSF as a diagnostic marker of PD.

## Introduction

Parkinson’s disease (PD), as the second most well-known neurodegenerative disorder, occurs in >55-years-old persons with ∼1% prevalence rate across the world, characterized by the progressive degeneration and loss of dopamine (DA) neurons and the formation and accumulation of Lewy bodies (LB) in the substantia nigra (SN) (Wang et al., 2017). PD is a complicated neurological disorder, caused by the interaction of many risk factors including pathogenic genes, environmental toxin, oxidative stress. The diagnosis of PD primarily depends on clinical symptoms including resting tremor, bradykinesia, muscle rigidity and postural gait instability. However, non-motor symptoms like REM behavior disorder, olfaction disorders, depression and gastrointestinal dysfunction develop inconspicuously long before the emergence of motor symptoms. Furthermore, a large amount of DA neurons (60∼70%) has already disappeared when occurrence and observation of these manifestations which seems too late for patients to benefit from current therapeutics. Therefore, it is essential to look for more feasible and objective diagnostic methods to recognize PD at the prodromal stage.

Recently, rising evidence imply that the miRNAs abundantly exist in a variety of body fluids with high stability and quantification as well as lower detection cost and shorter time, which make it a promising and potential biomarker (Marques et al., 2017; Wang et al., 2017; Lu et al., 2018). MiRNAs are a class of short-stranded, noncoding, endogenous RNAs consisting of 20–22 nucleotides. involved in the process of gene expression post-transcriptional regulation by combining their target genes at seed sequence of 3’-UTR (Singh and Sen, 2017). In fact, a large portion of miRNAs associated with or not with PD pathogenic genes were verified to regulate the expression of particular proteins participating in PD pathogenesis [(Wang et al., 2019), (Zhou et al., 2018)], which suggest that miRNAs play an important part in the pathogenesis of PD and may be a new potential therapeutic target of this disease (Lu et al., 2018). In addition, from most of surveys about PD candidate biomarkers, the results showed that the CSF closest to the brain is considered to be the best source to reflect the changes of molecular levels in the brain and should be more stable without well fluctuations due to the environmental changes than other body fluid (Marques et al., 2017; Taguchi and Wang, 2018; (Dos Santos et al., 2018).

Therefore, the goal of this study is to explore potential miRNAs as diagnostic indictors for early PD and influence of these miRNAs on the occurrence and development of PD. To obtain feasible miRNA indicators, the miRNA expression in CSF was quantified by the NG. Then, the miRNAs that have different expressions between PD patients at early stage and healthy controls were selected to be screened in the reported document database. If the miRNA has the same trend of the reported result, this miRNA will be identified as a feasible and ideal indicator of PD. Besides, to further study the function of the identified miRNAs, we discuss influences of the overexpression of miRNAs on apoptosis, which were detected in cell by TUNEL and Western Blot and in mice by immunofluorescence staining of brain sections, and the target gene of the identified miRNAs by dual luciferase reporter gene assay.

## Materials and Methods

### Clinical Samples and Patient Data

CSF was obtained by standard procedures from all subjects with written informed consent, recruited in the Department of Neurology, The second Xiangya Hospital, Central South University from July 2017 to June 2018. The inclusion criteria of early PD patients: 1) Patients meeting the 2015 clinical diagnosis criteria of MDS Parkinson’s disease and being jointly diagnosed by 2 senior neurology physicians (Postuma et al., 2015); 2) The onset age is 30 years old or above, and the duration of disease is less than 5 years; 3) Hoehn and Yahr (H&Y) stage is 2.5 or less. Exclusion criteria: 1) Patients were confirmed or suspected atypical Parkinson’s disease or drug-induced parkinsonism; 2) Score above 3 on Unified Parkinson’s Disease Rating Scale (UPDRS) in any limb; 3) MMSE score of 25 or less; 4) A history of stroke, intracranial space-occupying diseases, and infectious diseases of the nervous system; 5) A history of major trauma to the head or spinal cord or surgery; 6) Have been taking non-anti-Parkinson’s disease drugs for the past 3 months; 7) Lumbar puncture is not acceptable. The control group was collected from age-matched patients without neurodegeneration disease.

### RNA Isolation and Sequencing

The CSF was stored in polypropylene tubes placed at −80°C before further tested. The total RNAs were extracted using TRIzol reagent. The quantity and quality of RNA were checked by Nanodrop and Electrophoresis. The small RNA was added RNA 3' and 5' adapters at each end. Reverse transcription is used to create cDNA constructs based on the miRNA ligated with 3' and 5' adapters. A quality control analysis on sample library was performed by Agilent Technologies 2,100 Bioanalyzer using DNA-1000 or High Sensitivity DNA chip. Use cBot for the cluster generation and then sequence by Hiseq2500 (Illumina company, America).

### Cell Culture, Treatment and Transfection

SH-SY5Y cells were obtained from ATCC and cultured in the F-12 medium (Gibco, United States) supplemented with 10% fetal bovine serum (Hyclone, United States), 1% non-essential amino acid (NEAA), 1% glutamine, 1% sodium pyruvate and 100 U/mL penicillin and 100 μg/ml streptomycin (Gibco, United States). The cells were incubated at 37°C in a humidified chamber containing 5% carbon dioxide. SH-SY5Y cells were differentiated into neurons following cultured in F-12 medium with 10 μM retinoic acid, 10% FBS, 2 mM glutamine for 7 days, and further cultured in FBS-free F-12 medium with 50 ng/ml brain-derived neurotrophic factor (BDNF) (ThermoFisher, United States), 2 mM glutamine for 5 days. In the experiment, 1 mM MPP^+^ (Sigma, United States) were added to the cells. The overexpression of miRNA in the cells was by transfection with Lipofectamine 2000 reagent (Invitrogen, United States) according to the manufacturer’s protocol.

### RNAs Extraction, Reverse Transcription and Quantitative Real-Time PCR

Total small RNAs were isolated using TRIzol reagent (Invitrogen, United States) according to the manufacturer’s recommendations. Reverse transcription of all RNAs into cDNAs using the PrimeScript RT reagent Kit (ThermoFisher, United States), were performed at 16°C for 30 min, 42°C for 30 min and finally 85°C for 10 min. RT-qPCR to detect miRNAs expression were conducted at 95°C for 10 min to heat, followed by 40 cycles in the conditions: 95°C for 15 s to become denatured, 60°C for 30 s to annealing, then 72°C for 10 s to extend, with U6 as an internal control. Above PCR reactions were performed in triplicate. The primers were purchased in BioSune company (China). Our selected miRNAs have all been reported in previous publications. The sequence of primers used is shown in Supplementary Appendix S1.

### TUNEL Assay

The apoptosis rate of cells was measured using the TUNEL Kit (Sigma, United States) according to the manufacturer’s instructions. In this procedure, the SH-SY5Y cells were seeded on the coverslip placed on the 24-well plates (BD Falcon, United States) and then treated with MPP^+^ after being transfected with miRNA mimic (Ribo, China). The coverslips were washed twice with phosphate buffered saline (PBS), and fixed with 4% paraformaldehyde at 4°C for 30 min. After washed three times with PBS, the slices were covered with 50 μL TUNEL reaction mixture solution with deoxynucleotide fluorescein-12-dUTP and incubated at humidified atmosphere at 37°C for 60 min in the dark. After rinsing the slices with PBS for three times, the cells were treated with DAPI solution (Sigma, United States) shading at room temperature for 2 min. Finally, samples were rinsed twice with PBS, and slides were covered with mounting medium containing glycerin reagent (Sinopharm, China) and cover glasses (Sail Brand, China). Since then, the apoptotic cells were observed and photographed with a fluorescence microscope.

### Western Blot Analysis

Cells were lysed in buffer comprising 2% SDS, 62.5 mM Tris-HCl pH 6.8 and 10% glycerol. The tissues were homogenised in 10 volumes of RIPA buffer (Tris-HCl 50 mM, NaCl 150 mM, 1% Triton X-100, Sodium Deoxycholate 1%, SDS 0.1%, EDTA 2 mM). Protein extracts were measured by the bicinchoninic acid (BCA) quantification assay (Sigma, United States). Equal amounts of whole protein were electrophoresed on 15% SDS-polyacrylamide gel at constant voltage 80 V for 30 min and constant voltage 120 V for 40–50 min to separate the different Dalton proteins on the glue. Then appropriate protein was transferred to the polyvinylidene fluoride (PVDF) membranes (Millipore, United States) by wet type transfer apparatus (Bio-Rad, United States). After being blocked with 5% skim milk (Yili, China), the membranes were incubated with primary antibodies against human BAX, Bcl2 and cleaved caspase 3 (Cell Signaling Technology, United States) overnight at 4°C, and normalized to the endogenous β-actin (sigma life science, United States) as an endogenous control. Horseradish peroxidase (HRP) conjugated second antibodies against mouse or rabbit IgG (Cell Signaling Technology, United States) was used to incubate the blot for 1 h at room temperature. Finally, super signal chemiluminescence (ThermoFisher, United States) was used to detect the signal intensities.

### Animal Experiment

6 male B6 wild-type mice were raised under specific pathogen-free environment for 1 year. AAVs carried miRNA (Obio Technology, China) were injected to the substantia nigra pars compacta (SNc) of the mice brain through stereotaxic surgery. The coordinate axis parameter of SNc is ±1.25 mm in M/L, −3.16 mm in A/P, −4.25 mm in D/V. After 3 weeks, the MPTP (Sigma, United States) was administrated in these mice with injection every 2 h for four doses in total over an 8 h period (20 mg/kg per dose × 4). One-day interval later, these mice were sacrificed and their brains were removed. The brain tissues were fixed with 10% paraformaldehyde (Cell Signaling Technology, United States) for 1 day at room temperature, followed by dehydration in 30% sucrose for 2 days at 4°C and embedding with O.C.T.Compound (Solarbio, United States) at −80°C. The brain tissues were cut to 20 μm thick slices with the freezing microtome (Leica, Germany). The slices were blocked with 5% BSA powder (Proliant, United States) dissolved in PBS containing 0.3% Triton X-100 (Cell Signaling Technology, United States) for 1 h followed by incubation of rabbit tyrosine hydroxylase (TH) primary antibody (Abcam, United States, 1:200) overnight at 4°C and then with Fluor 594 (red) secondary antibody (ThermoFisher, United States) at 37°C for 30 min. Images were taken by Zeiss LSM 880 confocal microscope.

### Dual Luciferase Reporter Gene Assay

The target genes of miR-409-3p were predicted in a bioinformatics prediction website (http://www.targetscan.org) and were evaluated whether to directly target ATXN3 using a dual-luciferase reporter gene assay. The 3’-UTR of ATXN3 gene was inserted into XhoI and NotI restriction sites of pmiR-RB-REPORT™ vector. The recombinant sequence was confirmed by DNA sequencing. The miR-409-3p binding site mutation (GTTGTAAG change to GTCCAAAG) were constructed using Q5® Site-Directed Mutagenesis Kit (NEB, United States). The Dual Luciferase Reporter assay system used the renilla luciferase gene as the reporter luciferase and the firefly luciferase gene as an internal control.

### Immunofluorescence

The SH-SY5Y cells grown on coverslips were transfected with miRNA mimic (Ribo, China) and ATXN3-Q78-GFP.The cells were fixed with 4% paraformaldehyde for 15 min, permeabilized with 0.1% Triton X-100 in PBS for 15 min, and blocked with 5% fetal bovine serum (FBS) in PBS for 2 h at room temperature. Cells were then incubated with DAPI solution (Sigma, United States) shading at room temperature for 2 min. Finally, cells were mounted and visualized by confocal microscopy.

### Cell Apoptosis Detected by Flow Cytometry

The SH-SY5Y cells were seeded on the 24-well plates (BD Falcon, United States) and then treated with MPP^+^ for after being transfected with miRNA mimic (Ribo, China) and ATXN3-Q78-GFP. The apoptosis was detected by the Annexin V-FITC Apoptosis Detection Kit (Gibco, United States) according to the manufacturer’s protocol. The cells were trypsin-released and Annexin-V/PI stained at room temperature and kept in dark place for 5 min. Apoptotic cells were measured by flow cytometer (BD Bioscience, Accuro C6) and quantitated.

### Statistical Analysis

The profiling of miRNAs from CSF were constructed at Genergy company. Statistics from miRNA-Seq were performed and the expression of miRNAs was using Fold change (FC). Statistics from RT-qPCR were performed using Bio-Rad iQ5 software (Bio-Rad, United States). Relative expression of miRNA was calculated by REL = 2^−ΔΔCt^ (ΔΔCt= (Ct [experimental group]-C_t_ [U6]) -(C_t_ [experimental group]-C_t_ [U6])). Statistics from western blot were performed by the analysis of gray value at ImageJ software. Statistical analysis of data used GraphPad Prism v5.0 (CA, United States). Results of quantitative data in this research are expressed as mean ± SD. Significant differences in experiment between two groups were compared using Student *t*-test. A *p* value less than 0.05 was considered statistically significant (*p values < 0.05, **p values < 0.01, ***p values < 0.001; bar, SD).

## Results

### Variance Analysis of the Clinical Information

The demographic characteristics of the subjects in our study, including 7 early PD patients and 4 controls is summarized in Table 1. The PD group consisted of 3 males and 4 females, aged ranging from 44 to 59 years old with an average of 53 ± 3 years old, as well as their H&Y median is 2 and UPDRS III median: 24. 1 male and 3 females in the control group, aged 33–58 years old with average age of 46 ± 10. The difference about age, gender, and disease duration in the two groups is showed not statistically significant.

**TABLE 1:** Cohort summary.

	PD	Control	*p* value
Individuals (n)	7	4	NA
Gender (male in % (m/f))	43% (3/4)	25% (1/3)	NA
Age (in years mean +/− SD)	53 ± 5	46 ± 10	0.1479
Disease duration (in years mean)	2.5 ± 1.5	NA	NA
H&Y (median)	2 ± 0.7	NA	NA
UPDRS III (median)	24 ± 10.6	NA	NA

A total of 11 individuals were include in this study, 7 early stage PD, patients and 4 controls. Gender, age and disease duration were calculated for both groups and are presented above. H&Y = Hoehn and Yahr staging and UPDRS III = Unified Parkinson’s Disease Rating Scale. *p* values are calculated with Pearson Chi Square or *t*-test.

### miRNAs Profiling in CSF of Parkinson’s Disease Patients

**FIGURE 1:**
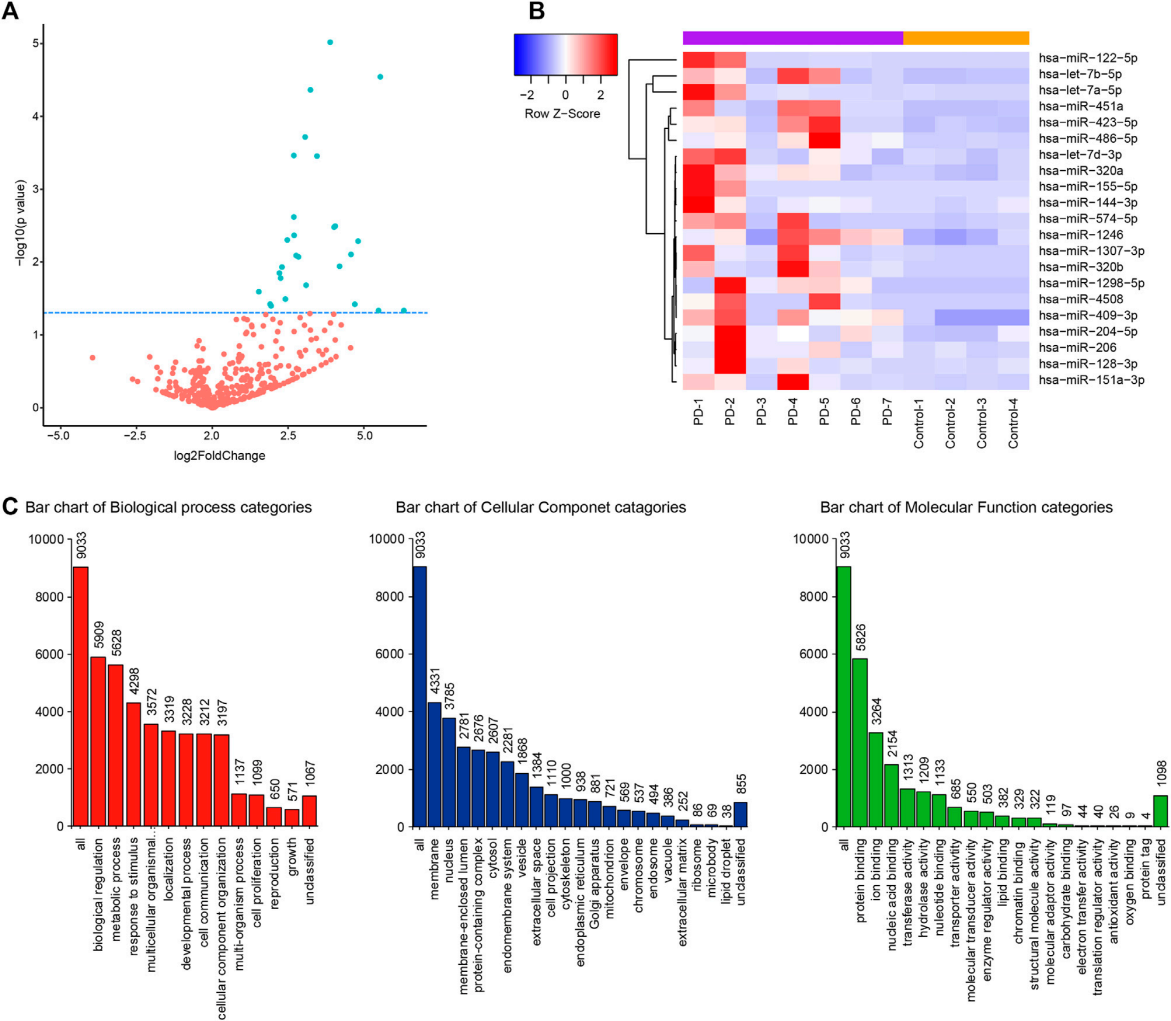
CSF miRNA profile data analysis and bioinformation prediction indicate the altered expression of miRNA in PD. **(A)** Volcano plot shows that most of altered CSF miRNAs were upregulated in PD patients compared to control group. Blue dots indicate a fold change expression > 1.5 [|log2 (Fold Change) | > 1] and *p* < 0.05 [−log10 (*p* value) > 1.3] **(B)**. Heatmap represents the mean fold change in PD and related differential miRNA signature. Each entry of the grid refers to relative fold (log2) between the expression level of a given miRNA in all participators. The color of each entry represents the abundance of miRNAs, ranging from blue (negative values) to red (positive values). **(C)**. GO enrichment analysis of predictive target genes of 21 different miRNAs in CSF between PD patients and control group.

The miRNA libraries from the CSF were analyzed on the Illumina HiSeq2500 platform. There were 21 unique miRNAs with significant difference (*p* < 0.05) between PD patients and the control group (Figures 1A,B; Table 2). Among them, all miRNAs were up-regulated with a fold change ≥ 1.5 (*p* < 0.05, Table 2). The target genes of 21 miRNAs were predicted in the website TargetScanHuman (http://www.targetscan.org). The total summarizing number of target genes is 9033 by wiping off the repetition. GO analysis respectively showed a proportion of these target genes in the biological process, cellular component and molecular function (Figure 1C and Supplementary Appendix S2). The signal pathway is involved in the mitochondrial function, electron transfer activity and antioxidant activity related to the pathogenesis of Parkinson’s disease.

**TABLE 2:** Top ranking variables.

Ranking	miRNA	*p* value	log2FoldChange
1	hsa-miR-486-5p	0.00000959	3.88993088
2	hsa-miR-122-5p	0.0000287	5.543851992
3	hsa-miR-451a	0.0000432	3.241350029
4	hsa-miR-423-5p	0.000192607	3.062007205
5	hsa-let-7b-5p	0.000343727	2.687544466
6	hsa-miR-151a-3p	0.000350179	3.452579624
7	hsa-miR-320a	0.002406471	2.690385725
8	hsa-miR-574-5p	0.003219922	4.058887315
9	hsa-miR-206	0.00331531	4.022111862
10	hsa-miR-204-5p	0.004298214	2.701213776
11	hsa-miR-1298-5p	0.005178984	2.47109027
12	hsa-miR-320b	0.007877432	4.807969959
13	hsa-miR-1246	0.008116925	4.573932171
14	hsa-miR-1307-3p	0.011427351	2.766017262
15	hsa-miR-128-3p	0.014178885	2.841556885
16	hsa-miR-409-3p	0.020788862	4.199636078
17	hsa-let-7a-5p	0.025565157	2.298103548
18	hsa-miR-144-3p	0.032294754	2.210960024
19	hsa-let-7d-3p	0.037862484	2.253885033
20	hsa-miR-4508	0.037949705	3.093618416
21	hsa-miR-155-5p	0.046302922	1.534609609

### Variation of the Selected miRNA in the Parkinson’s Disease Cell Model

**FIGURE 2:**
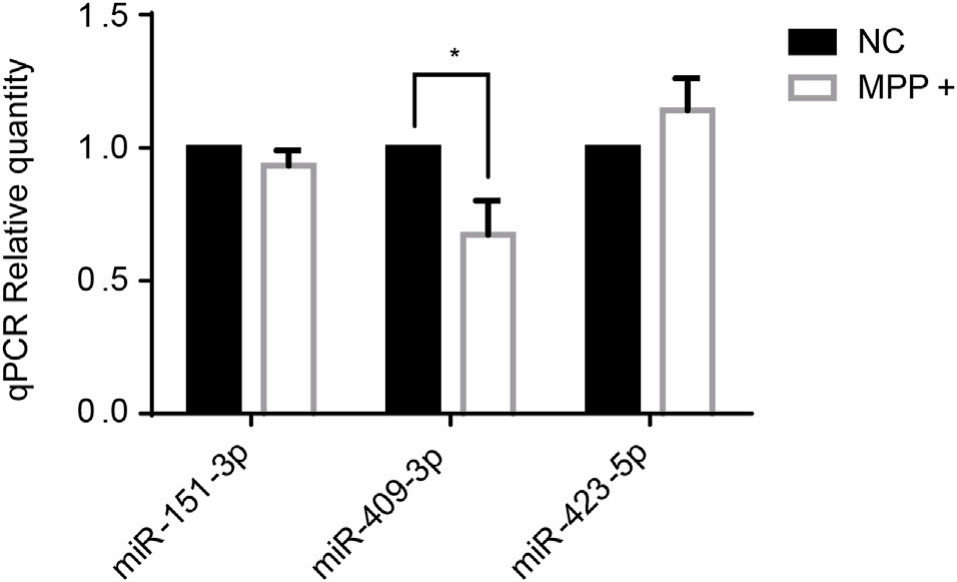
The expression level of miR-409-3p decreases significantly in MPP^+^-induced SH-SY5Y cells. The expression levels of three selected miRNAs were detected in PD model cells by RT-qPCR analysis. Data were analyzed using Student *t*-test, *p < 0.05.

Several miRNAs had been reported at abnormal level in PD, like miR-151-3p (Vallelunga et al., 2014), miR-409-3p ((Burgos et al., 2014), (Gui et al., 2015)), miR-423-5p ((Dos Santos et al., 2018), (Roser et al., 2018)) which were established in correlation with PD also in our study. Thus, the three miRNAs were selected to tested by RT-qPCR in SH-SY5Y cells treated with MPP^+^ *in vitro*. The results showed that miR-409-3p was decreased in the MPP^+^ treated cells (*p* < 0.05) (Figure 2).

### The Effect of Overexpression of miR-409-3p on Apoptosis in Cells Treatment of MPP^+^

**FIGURE 3:**
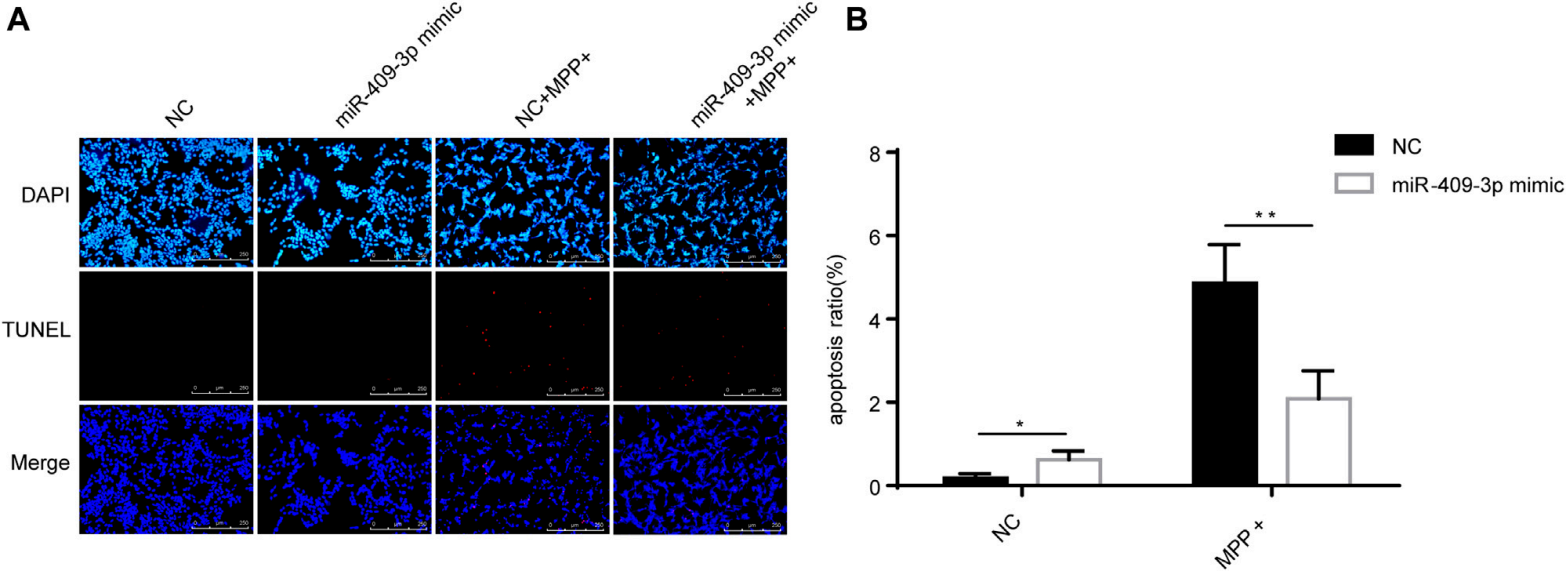
Overexpression of miR-409-3p inhibits apoptosis in MPP^+^-induced SY5Y cells. **(A)** TUNEL staining of SH-SY5Y cells apoptosis in each group. Scale bar = 50 μm. **(B)** Histogram shows SH-SY5Y cells apoptosis ratio decrease in the miR-409-3p mimic group. Data were analyzed using Student *t*-test, **p < 0.01.

**FIGURE 4:**
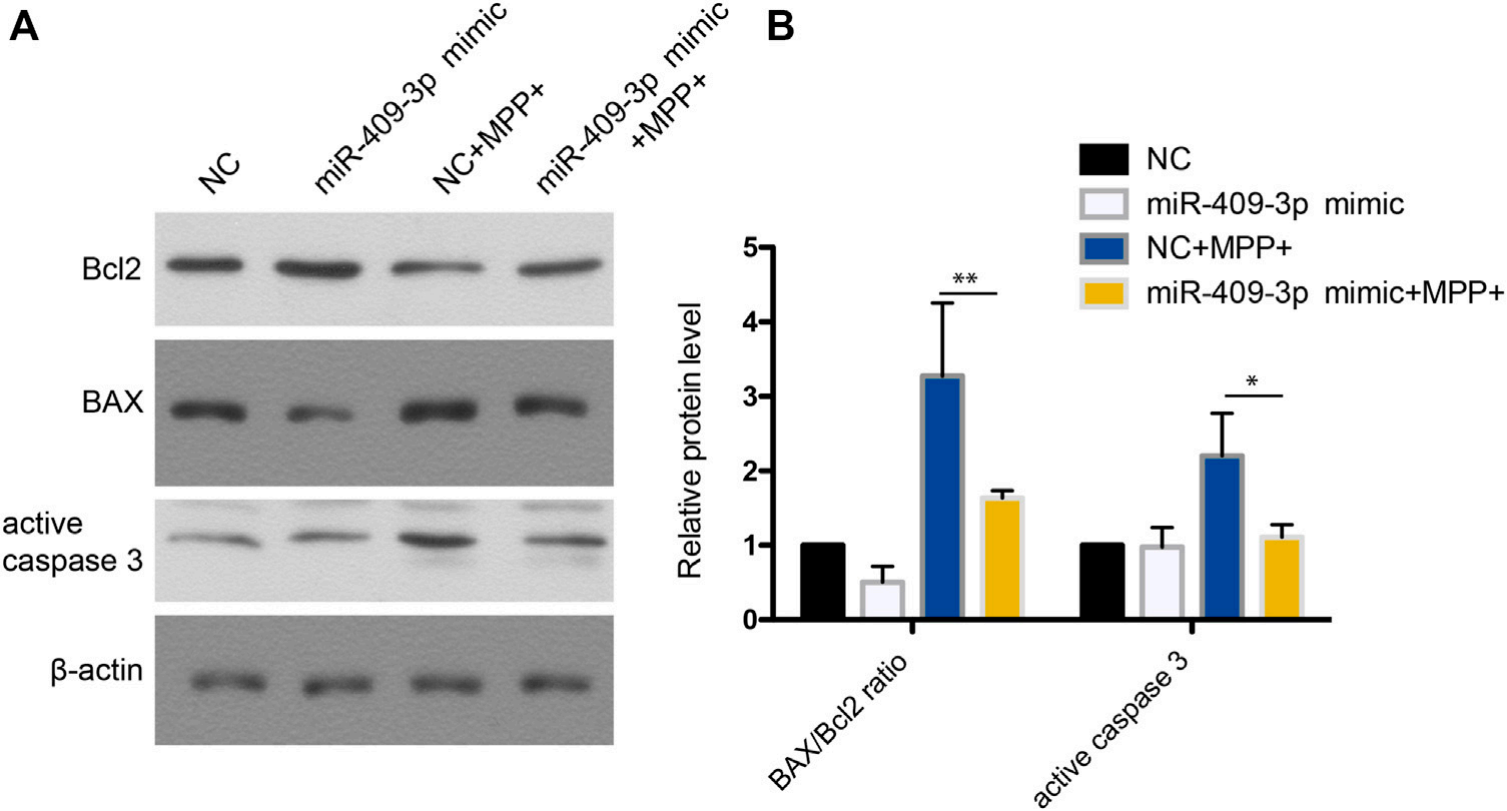
Western blot analysis shows that miR-409-3p overexpression inhibits apoptosis. **(A)** The apoptosis related proteins (BAX, Bcl2 and Cleaved caspase 3) were detected by western blot analysis in SH-SY5Y cells treated with MPP^+^ after miR-409-3p overexpression. **(B)** Histograms shows that the BAX/Bcl2 ratio and the level of active caspase 3 decrease in the miR-409-3p overexpression group with MPP^+^-induced apoptosis. Data were analyzed using Student *t*-test, *p < 0.05; **p < 0.01.

To investigate the roles of miR-409-3p in apoptosis induced by MPP^+^, miR-409-3p was overexpressed in the SH-SY5Y cell by transfecting the mimic into the cells over 24 h. The cells then were induced to apoptosis using the MPP^+^ for 48 h. The TUNEL analysis indicated that the apoptosis ratio reduced remarkably in the cells with overexpression of miR-409-3p, compared with control (Figure 3). Similarly, the western blot analysis revealed the BAX/Bcl2 ratio decreased and active caspase3 downregulated in miR-409-3p mimic group compared with control when simultaneously treated with MPP^+^ (Figure 4), which supports that overexpression of miR-409-3p can protect the apoptosis from MPP^+^ treatment.

### SNc Stereotaxic Injections for Overexpression of miR-409-3p *in vivo*

**FIGURE 5:**
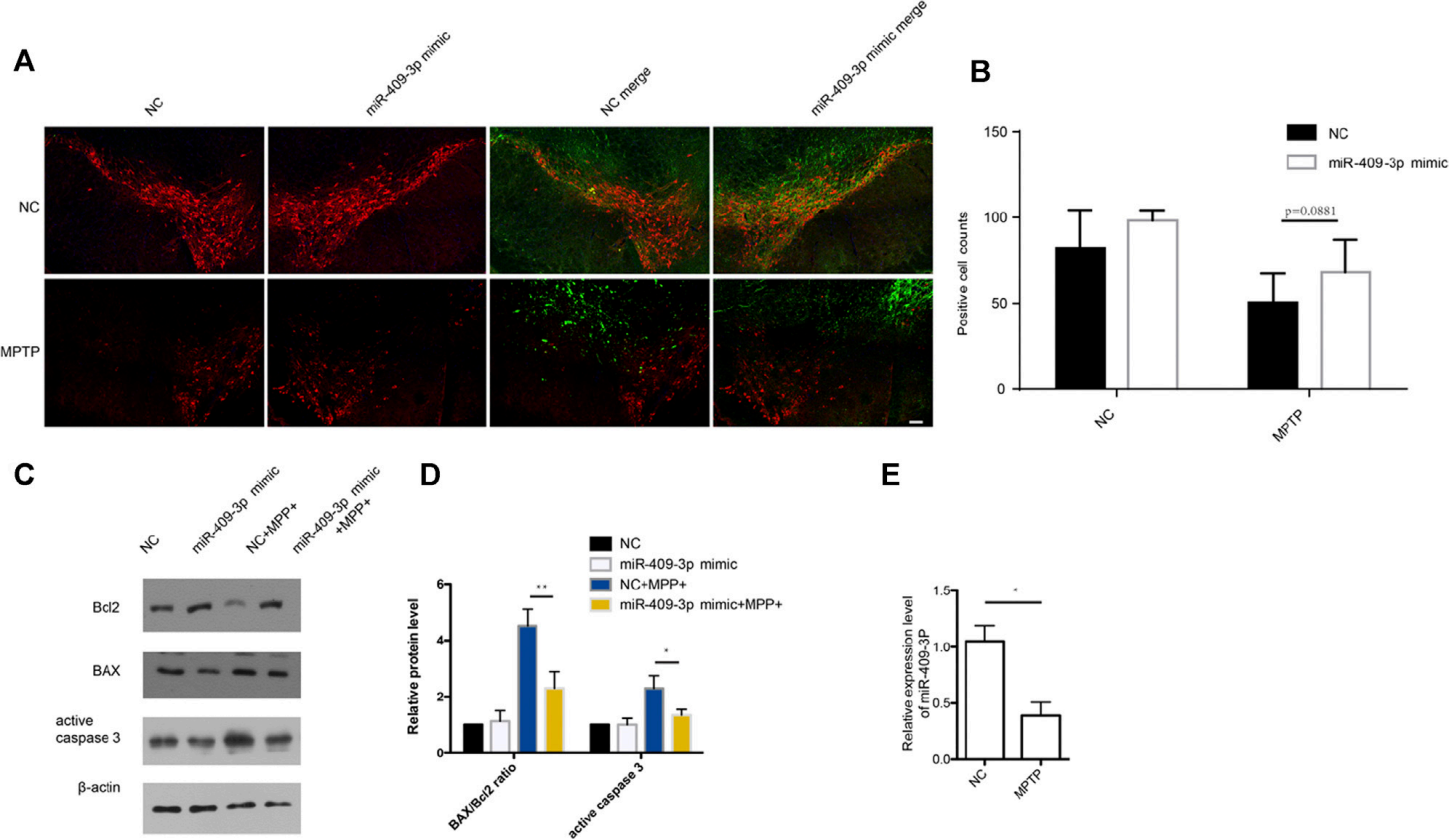
miR-409-3p overexpression protects loss of TH neurons induced by MPTP *in vivo*. **(A)** TH positive neurons (red) and miR-409-3p overexpression (green) in SNc were detected by immunofluorescence analysis. The mice were injected AAV carried with miR-409-3p control and mimic on the left and right sides of their brains. TH neuron numbers decrease after MPTP treatment. Scale bar = 50 μm. **(B)** Histograms used to count the number of TH cells shows a relatively amount of TH positive neurons at right side compared to control side. **(C)** The apoptosis related proteins (BAX, Bcl2 and Cleaved caspase 3) were detected by western blot analysis in SNc. **(D)** Histograms shows that the BAX/Bcl2 ratio and the level of active caspase 3 decrease in the miR-409-3p overexpression group with MPTP treatment. Student *t*-test, *p < 0.05; **p < 0.01. **(E)**. The expression of mouse miR-409-3p was detected by real-time PCR. Data were analyzed using Student *t*-test, *, *p* < 0.05.

We also tested the effect of miR-409-3p on MPTP-induced apoptosis of tyrosine hydroxylase (TH) neurons *in vivo*. The 12-months-age mice with overexpression of miR-409-3p through AAV infection in the SNc region of the right brain and control in the left for 3 weeks were then established the acute MPTP mouse model of PD. The immunofluorescence analysis indicated that a relatively denser distribution of TH positive neurons at the side of overexpression of miR-409-3p but rarely found at the control side. (Figures 5A,B). BAX/Bcl2 ratio and active caspase3 were also analyzed *in vivo*. The results showed BAX/Bcl2 ratio and active caspase3 were decreased in SNc region infected miR-409-3P mimic AAV comparing to SNc region infected with control AAV (Figures 5C,D). At the same time, we also detected a decrease of miR-409-3P in the SNc of mice treated with MPTP (Figure 5E).

### miR-409-3p Regulate Apoptosis-Related Protein Pathways by Targeting at *ATXN3*

**FIGURE 6:**
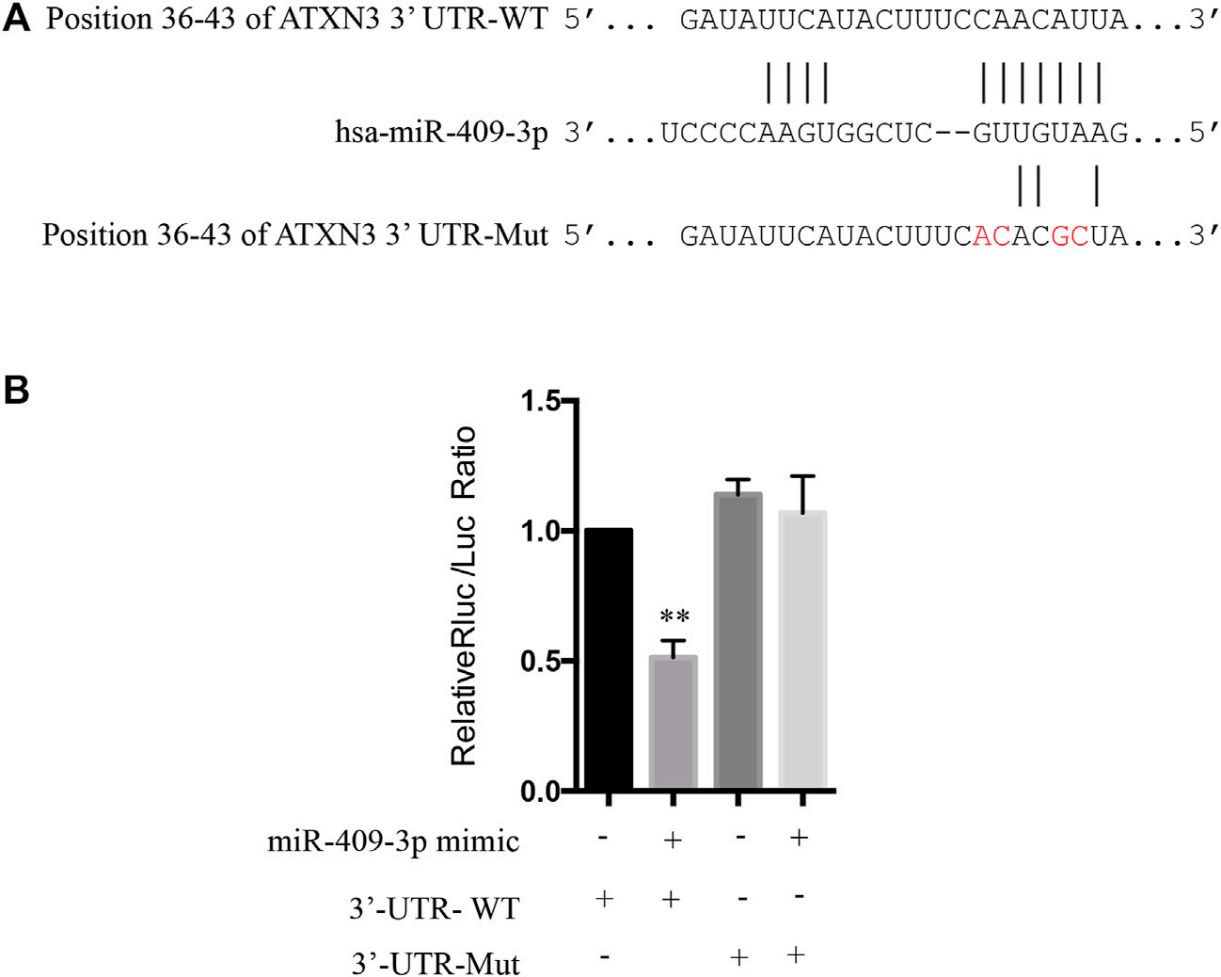
The prediction in bioinformatics and dual luciferase reporter gene assay to determine the targeted relationship between miR-409-3p and ATXN3. **(A)** The binding sites of miR-409-3p and the position 36-43 of ATXN3’ UTR-WT and ATXN3’ UTR Mut (GTTGTAAG change to GTCCAAAG) predicted in the website. **(B)** The relative luciferase activity of the cells co-transfected with miR-409-3p mimic and ATXN3-WT was significantly reduced. Data were analyzed using Student *t*-test, *p < 0.05; **p < 0.01.

**FIGURE 7:**
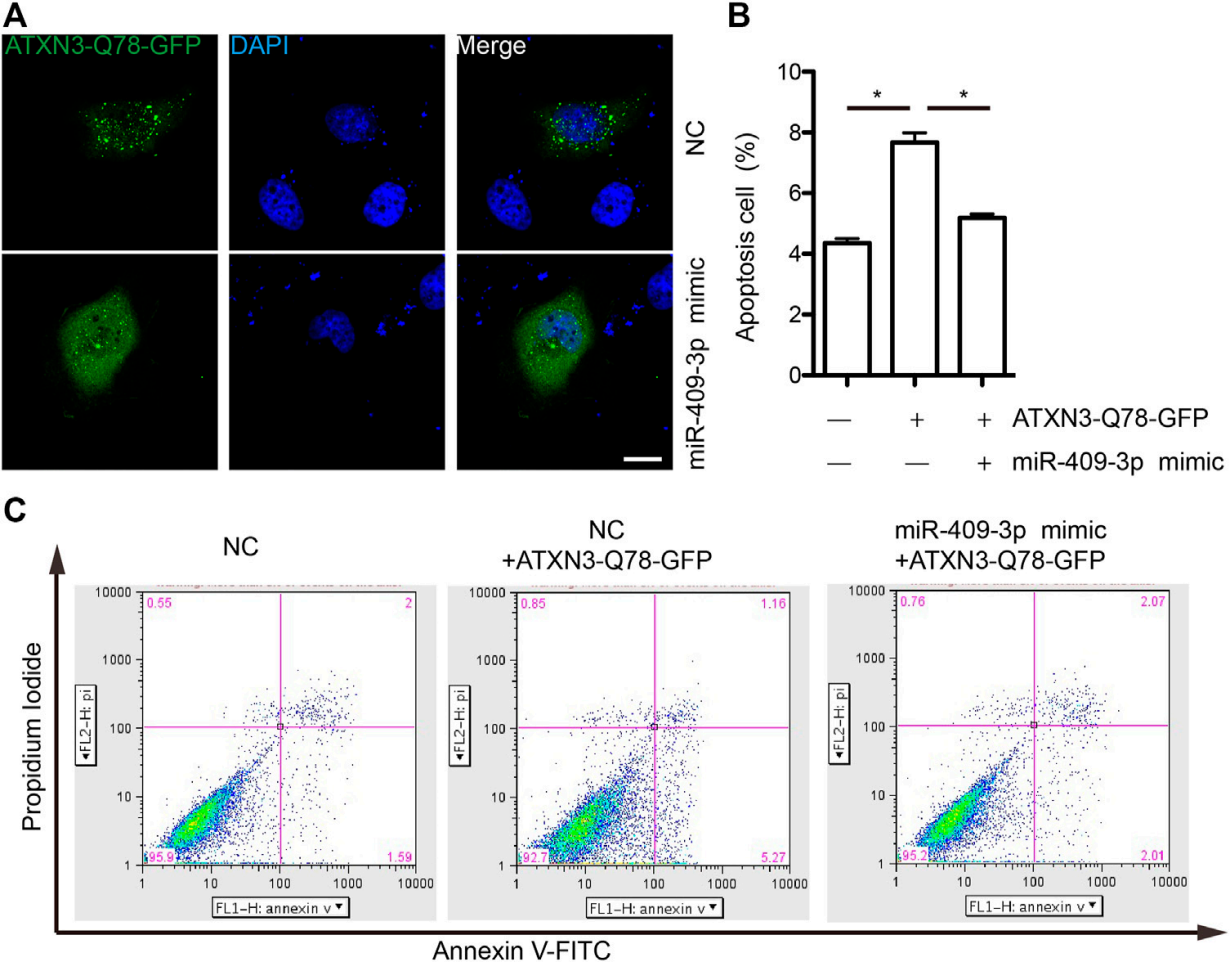
miR-409-3p decrease the cell toxicity of ATXN3-Q78-GFP **(A)** the aggregates of ATXN3-Q78-GFP were observed in SH-SY5Y cells with transfection of miR-409-3p mimic or negative control (NC). **(B)** Statistical results of apoptosis detected by flow cytometry in SH-SY5Y cells with transfection of miR-409-3p mimic or negative control (NC). Student *t*-test, *, *p* < 0.05. **(C)** Apoptosis were detected by flow cytometry in SH-SY5Y cells with transfection of miR-409-3p mimic or negative control (NC) using annexin-V-FITC and PI staining.

To explore the mechanism of miR-409-3p in neuroprotective effect, we analyzed the predicted miR-409-3p target genes that may affect mitochondrial function. ATXN3 is selected for further verification. miR-409-3p was verified to target the position 36–43 ATXN3 3’ UTR-WT (Figure 6A) using the online bioinformatics prediction website TargetScan (Figure 6A) and the dual luciferase reporter gene assay, which displays that the cells co-transfected with a miR-409-3p mimic and ATXN3-WT had lower relative luciferase activity than that of the NC group (*p* < 0.01), while the cells co-transfected with a miR-409-3p mimic and ATXN3-Mut showed no difference (Figure 6B). Both wild-type and polyglutamine-expanded mutants of ATXN3 have been reported to be involved in the mitochondrial function (Pozzi et al., 2008; Kristensen et al., 2018). Polyglutamine-expanded ATXN3 disrupts mitochondria by upregulating Bax and downregulating Bcl-xL and triggers apoptosis by inactivation of PNKP((Gao et al., 2015), (Chou et al., 2006)). To test the role of miR-409-3p in ATXN3-mediated apoptosis, we transfected miR-409-3p mimic in cells overexpressing ATXN3-Q78-GFP. The results show that miR-409-3p mimic can reduce the aggregates in ATXN3-Q78-GFP cells (Figure 7A). Moreover, miR-409-3p mimic can also decrease the apoptosis induced by ATXN3-Q78-GFP overexpression (Figures 7B,C).

## Discussion

PD has a prodromal period of about 20 years (Fereshtehnejad et al., 2019) but early PD has only about 65% diagnostic rate. Diagnosis error seems inevitable due to subjective judgment of symptoms and medical history without effective objective diagnostic criteria (Hustad et al., 2018). Therefore, many studies have been carried out to find different molecular markers (chemicals, metabolites, *etc*.) that are unique to PD patients from tissues to biofluids. With the expression of special conservation and stability and the ability of regulation of gene expression at post-transcriptional level, miRNAs become promising indicators for early diagnosis and target for treatment in future (Wang et al., 2017). In addition, it is generally believed that CSF is an ideal source reflect veritably the condition of neurodegenerative diseases, prior to other samples (Magdalinou et al., 2014).

### miRNAs Were Identified to Be a Feasible Indictor of Parkinson’s Disease

Altered microRNA profiles in CSF exosome in Parkinson disease are widely studied. Gui Y *et al* found miR-153, miR-409-3p, miR-10a-5p and let-7p-3p levels were increased significantly and miR-1 and miR-19b-3p levels were decreased in CSF by NGS and RT-PCR verification (Gui et al., 2015). miR-144-5p, miR-200a-3p, and miR-542-3p were found dysregulation in A53T mutant *α*-synuclein transgenic mice. These microRNAs were also shown significant increase in the CSF of PD patients (Mo et al., 2017). Additionally, our previous results showed the dysregulation of miR-626 in CSF were specific change in PD from other neurodegenerative diseases (Qin et al., 2019). A panel comprising 5 microRNAs (Let-7f-5p, miR-27a-3p, miR-125a-5p, miR-151a-3p, and miR-423-5p), with high sensitivity and specificity is a potential diagnostic tools for early stage Parkinson’s disease. Moreover, combined miRNA profiles with *α*-synuclein protein levels reach more high sensitivity and specificity (8). In our study, 21 miRNAs were identified at higher expression levels in CSF of PD by NGS (*p* < 0.05). The consistence of the change trend of miRNA expression was found in other studies. The expression level changes miR-423-5p and miR-151a-3p of in PD patients are consistent with the findings described above (Dos Santos et al., 2018). We also found the up-regulation of miR-409-3p in this study is similar to that of Burgos K *et al* (Burgos et al., 2014) and Starhof C *et al* (Starhof et al., 2019), which confirmed the high repeatability and stability of miRNA being a molecular marker for early diagnosis of PD.

### Influence of Up-Regulation of miRNAs on Parkinson’s Disease

As mentioned, miRNA may participate in PD related gene expression regulation and pathogenesis of PD. Therefore, miRNA expression changes can not only as diagnostic markers for PD, but also as new targets providing for the study of the emerging gene therapy. According to GO analysis, the predicted target genes of 21 miRNA play a role in a variety of biological processes and some are related to PD, which indicates that these abnormally expressed miRNAs may regulate the level of related proteins in the pathogenesis of PD.

Above three identified miRNAs were selected to be tested further in the PD cell model by qPCR. Among them, miR-409-3p showed a lower level in MPP^+^ induced SH-SY5Y cells, which contrary to the results observed in cerebrospinal fluid. There may be a negative feedback regulation of miRNAs in the human body and some studies have found that miRNA target genes can lower the expression of miRNAs by influencing miRNA promoters (O'Brien et al., 2018). Therefore, we speculate that the opposite trend of miR-409-3p is because the down-regulated signal of miR-409-3p in the patient at the beginning of the disease will mobilize the body’s negative feedback regulation mechanism to cause miR-409-3p increase in CSF.

Usually, miR-409-3p was previously reported to be associated with cancer such as cervical cancer (Shukla et al., 2019), breast cancer (Ma et al., 2016) and colorectal cancer (Bai et al., 2015). However, in the results of TUNEL, it was found that the overexpression of miR-409-3p can reduce the apoptosis of SH-SY5Y cells induced by MPP^+^. Therefore, to explore its function in PD, we predicted the target genes of human miR-409-3p and mouse miR-409-3p, respectively. Many of their target genes overlap. Some of these target genes involve mitochondria and apoptosis, such as *ATXN3* (ataxin 3), which play a activate role in the pathway of mitochondrial autophagy by upregulating the ratio of apoptosis-related protein Bax/Bcl-XL (Chou et al., 2006). Consistent with the result of WB, it was found that miR-409-3p affect the expression levels of apoptosis-related proteins BAX/Bcl2 and active caspase 3. We also found the overexpression of miR-409-3p in the brain of the mice can protect from the damage of toxicology. Furthermore, our dual luciferase reporter gene assay verified the miR-409-3p binding to *ATXN3.*

### Future Work

Our findings provided evidence that miR-409-3p plays an important role in PD and probably have a major impact on the diagnosis and treatment of the disease. Future research needs to validate miR-409-3p as an early diagnosis of PD in a larger population. Research on the regulation mechanism of disease-specific miRNAs will also help us understand the pathogenesis of PD.

## Conclusion

Our study further indicates that miRNAs in CSF can be ideal biological biomarkers for PD and it is valuable to be explored its function. Above all, the miR-409-3p seem to act a significant part in PD.

## Data Availability

The raw sequencing data that support the findings of this study have been deposited and released in the Sequence Read Archive under the BioProject accession number: PRJNA787975.
