# Supplementary material for: MicroRNA-409-3p Targeting at ATXN3 Reduces the Apoptosis of Dopamine Neurons Based on the Profile of miRNAs in the Cerebrospinal Fluid of Early Parkinson’s Disease
Source: Front Cell Dev Biol. 2022 Jan 10;9:755254. doi: 10.3389/fcell.2021.755254 (PMC8803123; doi:10.3389/fcell.2021.755254)
Supplement: Supplementary file 1 [file DataSheet1.zip › Appendixes/Appendix 1.docx]

| Name | RT-primer | F-primer | R-primer |
| --- | --- | --- | --- |
| miR-409-3p | GTCGTATCCAGTGCAGGGTCCGAGGTATTCGCACTGGATACGACAGGGGT | CACGCAGAATGTTGCTCGG | CCAGTGCAGGGTCCGAGGTA |
| miR-151a-3p | GTCGTATCCAGTGCAGGGTCCGAGGTATTCGCACTGGATACGACCCTCA | CACGCACTAGACTGAAGCTCC | CCAGTGCAGGGTCCGAGGTA |
| miR-423-5p | GTCGTATCCAGTGCAGGGTCCGAGGTATTCGCACTGGATACGACAAAGTC | CACGCATGAGGGGCAGAG | CCAGTGCAGGGTCCGAGGTA |
| U6 | Random primers | CTCGCTTCGGCAGCACA | AACGCTTCA CGAATTTGCGT |

**Appendice 1: The sequence of primers**
