# Supplementary material for: MicroRNA-409-3p Targeting at ATXN3 Reduces the Apoptosis of Dopamine Neurons Based on the Profile of miRNAs in the Cerebrospinal Fluid of Early Parkinson’s Disease
Source: Front Cell Dev Biol. 2022 Jan 10;9:755254. doi: 10.3389/fcell.2021.755254 (PMC8803123; doi:10.3389/fcell.2021.755254)
Supplement: Supplementary file 1 [file DataSheet1.zip › Appendixes/Appendix 2.docx]

| **Appendice 2: GO enrichment analysis of 21 miRNA** | | | |
| --- | --- | --- | --- |
| Classification | Pathway Name | GO.ID | Genes Regulated by miRNAs |
| Biological process | biological regulation | GO:0065007 | 5909 |
|  | metabolic process | GO:0008152 | 5628 |
|  | response to stimulus | GO:0050896 | 4298 |
|  | multicellular organismal process | GO:0032501 | 3572 |
|  | localization | GO:0051179 | 3319 |
|  | developmental process | GO:0032502 | 3228 |
|  | cell communication | GO:0007154 | 3212 |
|  | cellular component organization | GO:0016043 | 3197 |
|  | multi-organism process | GO:0051704 | 1137 |
|  | cell proliferation | GO:0008283 | 1099 |
|  | reproduction | GO:0000003 | 650 |
|  | growth | GO:0040007 | 571 |
| Cellular Componet | membrane | GO:0016020 | 4331 |
|  | nucleus | GO:0005634 | 3785 |
|  | membrane-enclosed lumen | GO:0031974 | 2781 |
|  | protein-containing complex | GO:0032991 | 2676 |
|  | cytosol | GO:0005829 | 2607 |
|  | endomembrane system | GO:0012505 | 2281 |
|  | vesicle | GO:0031982 | 1868 |
|  | extracellular space | GO:0005615 | 1384 |
|  | cell projection | GO:0042995 | 1110 |
|  | cytoskeleton | GO:0005856 | 1000 |
|  | endoplasmic reticulum | GO:0005783 | 938 |
|  | Golgi apparatus | GO:0005794 | 881 |
|  | mitochondrion | GO:0005739 | 721 |
|  | envelope | GO:0031975 | 569 |
|  | chromosome | GO:0005694 | 537 |
|  | endosome | GO:0005768 | 494 |
|  | vacuole | GO:0005773 | 386 |
|  | extracellular matrix | GO:0031012 | 252 |
|  | ribosome | GO:0005840 | 86 |
|  | microbody | GO:0042579 | 69 |
|  | lipid droplet | GO:0005811 | 38 |
| Molecular Function | protein binding | GO:0005515 | 5826 |
|  | ion binding | GO:0043167 | 3264 |
|  | nucleic acid binding | GO:0003676 | 2154 |
|  | transferase activity | GO:0016740 | 1313 |
|  | hydrolase activity | GO:0016787 | 1209 |
|  | nucleotide binding | GO:0000166 | 1133 |
|  | transporter activity | GO:0005215 | 685 |
|  | molecular transducer activity | GO:0060089 | 550 |
|  | enzyme regulator activity | GO:0030234 | 503 |
|  | lipid binding | GO:0008289 | 382 |
|  | chromatin binding | GO:0003682 | 329 |
|  | structural molecule activity | GO:0005198 | 322 |
|  | molecular adaptor activity | GO:0060090 | 119 |
|  | carbohydrate binding | GO:0030246 | 97 |
|  | electron transfer activity | GO:0009055 | 44 |
|  | translation regulator activity | GO:0045182 | 40 |
|  | antioxidant activity | GO:0016209 | 26 |
|  | oxygen binding | GO:0019825 | 9 |
|  | protein tag | GO:0031386 | 4 |
